# 

**Published:** 1873-01

**Authors:** 


VOL. III.] JANUARY, 1873. [No. ~~8.~~ 1

### THE SOUTHERN No. I.

### MEDICAL RECORD:

A MONTHLY JOURNAL OF

### PRACTICAL MEDICINE.

EDITED BY

T. S. POWELL, M.D.,

AND

W. T. GOLDSMITH, M. D.

*"QUICQUID PRÆCIPIES ESTO BREVIS."*

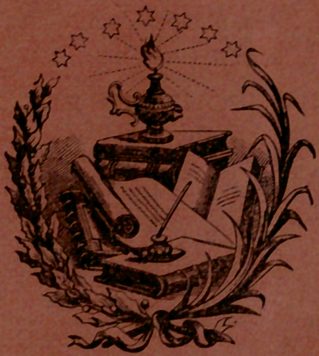

ATLANTA, GEORGIA:
PLANTATION PUBLISHING COMPANY'S PRESS.
1873.

TERMS, $2 50 PER ANNUM, IN ADVANCE. SINGLE NUMBER, 25 CENTS.

